# Mindfulness-Based Programs: Why, When, and How to Adapt?

**DOI:** 10.1177/21649561211068805

**Published:** 2022-01-27

**Authors:** Eric B. Loucks, Rebecca S. Crane, Menka A. Sanghvi, Jesús Montero-Marin, Jeffrey Proulx, Judson A. Brewer, Willem Kuyken

**Affiliations:** 1 Department of Epidemiology, Brown University School of Public Health, Providence, RI, USA; 2 Department of Behavioral and Social Sciences, 1506 Brown University School of Public Health, Providence, RI, USA; 3 Mindfulness Center, Brown University, Providence, RI, USA; 4 Centre for Mindfulness Research and Practice, School of Psychology, Bangor University, Bangor, UK; 5 174610 The Mindfulness Initiative, Sheffield, UK; 6 Department of Psychiatry, 6752 Oxford University, Oxford, UK; 7 Oxford Mindfulness Centre, 6396 Oxford University, Oxford, UK

**Keywords:** mindfulness, study design, implementation, dissemination

## Abstract

This paper provides a framework for understanding why, when and how to adapt mindfulness-based programs (MBPs) to specific populations and contexts, based on research that developed and adapted multiple MBPs. In doing so, we hope to support teachers, researchers and innovators who are considering adapting an MBP to ensure that changes made are necessary, acceptable, effective, cost-effective, and implementable. Specific questions for reflection are provided such as (1) Why is an adaptation needed? (2) Does the theoretical premise underpinning mainstream MBPs extend to the population you are considering? (3) Do the benefits of the proposed adaptation outweigh the time and costs involved to all in research and implementation? (4) Is there already an evidenced-based approach to address this issue in the population or context? Fundamental knowledge that is important for the adaptation team to have includes the following: (1) essential ingredients of MBPs, (2) etiology of the target health outcome, (3) existing interventions that work for the health outcome, population, and context, (4) delivery systems and settings, and (5) culture, values, and communication patterns of the target population. A series of steps to follow for adaptations is provided, as are case examples. Adapting MBPs happens not only by researchers, but also by MBP teachers and developers, who endeavor to best serve the populations and contexts they work within. We hope that these recommendations for best practice provide a practical framework for skilfully understanding why, when, and how to adapt MBPs; and that this careful approach to adaptation maximizes MBP safety and efficacy.

## Introduction

Case Example 1*:* Alejandro Zima is a bilingual Spanish/English Licensed Mental Health Counselor and Mindfulness-Based Stress Reduction (MBSR) teacher. He specializes in grief counseling. Alejandro was considering whether to offer MBSR in a hospice setting to family members of hospice patients, particularly in their second year of grief or later as a “step beyond” program. His hospice setting already offered effective psychoeducational and counseling programs for the first year of grief (some that included basic mindfulness training) but there was a gap in effective programs beyond that time. In fact, most participants in his MBSR programs had participated in prior grief support programs and/or were currently receiving ongoing counseling supports. Informed by his training in MBSR, counseling theory, grief support methodologies, trauma-sensitive mindfulness, and post-traumatic growth, Alejandro felt that MBSR would likely be effective, but with a few tailored considerations in the participant screening process and curricular modifications, to help it better support people who are healing following the passing of a loved one.

Case Example 2: Eric Loucks, a cardiovascular epidemiologist and MBSR teacher, was interested in whether MBSR might reduce risk for cardiovascular disease. Turning to the scientific literature, the effects of MBSR in a systematic review and meta-analysis showed reductions of systolic blood pressure of 6.6 (95% CI: −11.7, −1.5) mmHg at immediate post-intervention follow-up.^1^ Long-term effects are not yet clear.^1^ He already knew research showed that major drivers of blood pressure are diet, physical activity, obesity, excessive alcohol consumption, and antihypertensive medication adherence, which MBSR does not explicitly address other than through yoga training.^2^ He wondered if MBSR’s effects could be boosted, and MBSR be more accepted by people with hypertension, if its curriculum more explicitly directed mindfulness skill development to participants’ health behaviors that affect blood pressure (detailed elsewhere).^3^

Case example 3. Mark Williams, a clinical psychologist was interviewed about Mindfulness-Based Cognitive Therapy (MBCT) for depression by a journalist, Danny Penman, for an article in a UK national newspaper. They got to talking about whether MBCT might go beyond depression to help all of us who are struggling with the everyday demands and challenges of life, including whether it could support well-being in the wider population. They collaborated on answering these questions and wrote a book, *Mindfulness – A Practical Guide to Finding Peace in a Frantic World,* that has been read by more than 1.5 million people and translated into more than 30 languages.^4^ The work has been developed, implemented and researched as an in-person course.^5,6^

There has been an explosion of interest in mindfulness-based programs (MBPs).^7-9^ MBSR, which teaches mindfulness practices in group settings over an 8-week period using an established curriculum, has undergone hundreds of randomized control trials from which systematic reviews and meta-analyses show effects on outcomes such as stress, anxiety symptoms, depressive symptoms and chronic pain management.^7-11^ MBCT integrates MBSR with cognitive behavioral therapy (CBT) in ways that target the vulnerability factors for people at risk of recurrent depression. MBCT and MBSR have increasingly demonstrated effectiveness and cost-effectiveness, providing millions of people with choices about how best to recover and stay well in the longer term.^8,12^

Partially because of this careful work to develop and research MBSR and MBCT, there is growing awareness of, and demand for, MBPs in new populations and contexts. This has led to a proliferation of MBP innovations with potential to improve outcomes in target populations. However, there are also costs and downsides to adaptations. Careful adaptation is time and resource intensive. It requires research to investigate the acceptability, effectiveness and cost-effectiveness, and whether the adaptations work through hypothesized mechanisms. A key question is whether adaptations add value to other already available evidence-based programs. Finally, even if the adaptation proves to be acceptable and cost-effective, real-world implementation is complex and time consuming (such as training teachers, developing digital platforms and persuading policy groups to recommend it). Theory and research point to the facilitators and barriers involved in navigating an evidence-based MBP to targeted populations and new contexts.^13-16^

Nonetheless, there are several excellent examples of MBPs adapted to specific populations and contexts, such as depression (MBCT),^8^ binge eating disorder (Mindfulness-Based Eating Awareness Training),^17^ relapse prevention (Mindfulness-Based Relapse Prevention),^18^ cancer (Mindfulness-Based Cancer Recovery),^19^ and blood pressure (Mindfulness-Based Blood Pressure Reduction).^20^ There are increasing adaptations to specific *demographic groups,* such as young adults (Mindfulness-Based College),^21^ military personnel (Mindfulness-Based Mind Fitness Training, Mindfulness-Based Attention Training),^22^ those with trauma history,^23^ expectant parents (Mindfulness-Based Childbirth and Parenting Program),^24^ and Native American communities (NativeMIND).^25^ There are many more, and the evidence base and penetration of these MBPs varies greatly, from early preliminary studies (e.g., NativeMIND), through to extensive evidence and implementation around the world (MBCT). In some cases, implementation far outstrips the evidence, for example, with some mainstream mindfulness apps that are used by millions internationally. There are early promising findings for adaptations to online delivery formats: for example, randomized controlled trial showed online MBCT effectively prevents depression relapse^26^; a pilot single-arm trial show the Eat Right Now app improved emotional eating^27^; a systematic review of face-to-face MBPs delivered through videoconferencing online suggests beneficial effects.^28^

A growing number of resources are available to guide and support MBP adaptation. These include established best practice methods for developing behavioral change interventions (Table 1), including the Science of Behavior Change framework,^29^ MRC Guidelines on Complex Intervention Development,^30^ Theory of Change,^31^ National Institutes of Health Stage Model,^16^ Multiphasic Optimization Strategy (MOST),^32^ amongst others. The Fieldbook for Mindfulness Innovators is a resource for making minor adaptations to existing MBPs, through to creating completely new MBPs.^33^ It emphasizes design thinking, prototyping, and offers steps for building evidence. Some textbooks describe examples of MBPs that have been adapted to specific populations and contexts.^34,35^ Dobkin et al. provide recommendations on staying true to core MBSR teaching intentions and program components, when adapting for specific populations and contexts.^36^ In response to the proliferation in the field, some of the first- and second-generation MBP developers created a consensus statement that describes the essential theoretical and curriculum elements of MBPs, and was written to support the sustainable development of the field.^37^ This paper, entitled *What Defines Mindfulness-Based Programs? The Warp and the Weft,* outlines both MBPs’ essential elements (the warp) and the flexible elements that can be adapted for particular populations and contexts (the weft) (Table 2).

**Table 1.** Established Models for Behavioral Intervention Development.

Model	Description
NIH Stage Model^15,16^	• Strong emphasis on carrying behavioral clinical trials through all clinical trial stages, including basic research for intervention development, research on mechanisms, efficacy and effectiveness testing, as well as implementation and dissemination research in the actual communities and settings the intervention ends up serving
Science of Behavior Change (SOBC)^29^	• Focuses on identifying mechanisms of behavior change first as an early indicator of effect, and as a potential target to customize interventions to engage with
	• Emphasizes evaluating the degree to which changes in the mechanisms translate into meaningful behavior change
	• Can foster creating efficient interventions customized to target the mechanisms of behavior change, while cleaving out superfluous intervention content that does not impact health
ORBIT Model^38^	• Incorporates basic behavioral and social science insights into a four-stage model of sequential intervention development and testing from phase I (intervention design) to phase IV (intervention effectiveness) testing
MRC Guidelines on Complex Intervention Development^30^	• Provides a framework for complex behavioral intervention development
	*A. “Pre-Clinical” or Theoretical Phase:* Identify theory to ensure best choice of intervention and hypotheses, and pinpoint confounders and challenges in intervention design
	*B. Phase 1 or Modeling:* Identify components of the intervention, and the underlying mechanisms by which they influence health outcomes, in order to predict how the components are related to, and interact with, each other
	*C. Phase 2 or Exploratory Trial:* Describe the constant and variable components of an intervention that should be replicable, and a protocol for comparing the feasibility of the intervention to an appropriate control
	*D. Phase 3 or Main Trial:* Compare the well-defined intervention to an appropriate control, using a theoretically sound, reproducible, methodologically rigorous protocol
	*E. Phase 4 or Long-Term Surveillance:* Determine whether others can reliability replicate the intervention and results in controlled settings over the long-term
Theory of Change (ToC)^31^	• Interventions developed in collaboration with a wide variety of stakeholders
	• Encompasses strategic considerations such as including beneficiaries, actors in the context, sphere of influence, research evidence supporting the ToC, timelines, and indicators
	• Emphasizes developing the theory by which the intervention is expected to change clinically relevant outcomes. By deeply understanding and developing the theory through recursive feedback from key stakeholders and scientific findings, it argues that more efficient and effective interventions can be developed
Community-Based Participatory Research (CBPR)^39^	• Focuses on active involvement of community members, organizational representatives, and researchers in the entire research process
	• Several key principles, identified by Israel et al., 52 are
	A. Recognizes community as a unit of identity
	B. Builds on strengths and resources within the community
	C. Facilitates collaborative partnerships in all phases of the research
	D. Integrates knowledge and action for mutual benefit of all partners
	E. Promotes a co-learning and empowering process that attends to social inequalities
	F. Involves a cyclical and iterative process
	G. Addresses health from both positive and ecological perspectives
	H. Disseminates findings and knowledge gained to all partners
Multiphasic Optimization Strategy (MOST)^32^	• Uses a three-phase design to identify the active and inactive components of interventions in order to make them efficient and effective. The phases are
	*A. Preparation*, in which a conceptual model is created, and pilot testing of intervention components is performed. Careful consideration is given to balancing effectiveness, affordability, scalability, and efficiency (EASE)
	*B. Optimization*, where the investigators select the components and component levels within the intervention, often using an optimization trial
	*C. Evaluation*, using a randomized controlled trial comparing the optimized interventions to an appropriate control group

**Table 2.** Description of Essential (Warp) and Flexible (Weft) Ingredients of MBPs and MBP Teachers Adapted From Crane et al.^37^

Warp: Essential ingredients	
MBP	MBP teacher
1. Is informed by theories and practices that draw from a confluence of contemplative traditions, science, and the major disciplines of medicine, psychology and education	1. Has particular competencies which enable the effective delivery of the MBP.
2. Is underpinned by a model of human experience which addresses the causes of human distress and the pathways to relieving it	2. Has the capacity to embody the qualities and attitudes of mindfulness within the process of the teaching
3. Develops a new relationship with experience, characterized by present moment focus, decentering and an approach orientation (i.e., moving towards experience—whether pleasurable, neutral or difficult—instead of away)	3. Has engaged in appropriate training and commits to ongoing good practice
4. Engages the participant in a sustained intensive training in mindfulness meditation practice, in an experiential inquiry-based learning process and in exercises to develop insight and understanding	4. Is part of a participatory learning process with their students, clients or patients
Weft: Flexible ingredients	
**MBP**	**MBP teacher**
1. The core essential curriculum elements are integrated with adapted curriculum elements, and tailored to specific contexts and populations	1. Has knowledge, experience and professional training related to the specialist populations that the mindfulness-based course will be delivered to
2. Variations in program structure, length and delivery are formatted to fit the population and context	2. Has knowledge of relevant underlying theoretical processes which underpin the teaching for particular contexts or populations

This paper is a sequel to the warp and weft paper, providing a detailed framework on why, when and how to adapt MBPs to specific populations and contexts. Currently, such a framework for MBP adaptation is absent in the literature. We start by addressing the obvious first questions: “Why is an adaptation necessary?” “When should I adapt, and when shouldn’t I?” We then go on to address the question, “How should I adapt for this population or context?” Our intention is to support MBP teachers (case example 1), researchers (case example 2) and innovators (case example 3) in considering whether and how to adapt MBPs. In doing so, we hope to support participants and the wider field by ensuring MBP teaching is acceptable, effective, cost-effective, implementable, and underpinned by best professional ethical codes and practices. While considering adapting MBPs, we encourage qualities such as humility, curiosity, open-mindedness, clarity about aims, embracing diverse and challenging voices, listening, testing, iterating, improving, and continued engagement with personal mindfulness practice. In these ways, we anticipate that adaptations will have the greatest likelihood of being safe and helpful.

## Why and When to Adapt an MBP?

Any MBP taught by a skillful teacher is continually being tailored and responsively tuned moment-by-moment to the individual, group and context. Teachers are always formulating and reformulating what is needed to support participants’ learning, fine-tuning their teaching to ensure it is inclusive and supports individual participants and the needs of the whole group.^40^ For example, stress or depression are experienced in both similar and unique ways, and good MBP teaching accommodates this universality and specificity. The questions in Table 3 critically analyze why and when to go beyond these expected adaptations to more systematically adapt an MBP to a particular context or population.

**Table 3.** Five Questions for Reflection in Considering Whether or Not to Adapt an MBP.

Question	Description
1. Why is an adaptation needed? Does a current MBP not meet the population being served? For example, is the adaptation minor (i.e., within the remit of the teacher’s ability to dial up/down certain features of an extant MBP for the population/ context) or is the necessary adaptation more major, and so requires the MBP curriculum and/or teaching process itself to be adapted?	• MBSR teachers offer MBSR in numerous settings and populations. An extensive evidence-base attests to MBSR’s acceptability and effectiveness^1^
	• Case example 1 of teaching MBSR in a hospice setting illustrates these questions of flex or adaptation. There is a substantial evidence-base showing MBSR reduces stress and improves mental health.^4^ Alejandro reasoned, informed by his clinical experience and the literature, that the practice of mindfulness during grief recovery could create space for careful observation, self-awareness and compassionate engagement with a griever’s varied emotions.^2^ Giving full attention to the griever’s own emotional state may allow for greater acceptance of these emotional ongoing changes as an important, though challenging, part of the grief process. Overall, he discerned these practices could lead to more effective integration of the sometimes overwhelming internal and external shifts associated with loss. In Alejandro’s hospice setting, the people he considered for taking the MBSR program were family members of hospice patients who had already participated in other individual or group grief counseling programs, had concurrent psychosocial supports, had participated in shorter mindfulness-based support sessions or had their own previous mindfulness practice, were at least 9 months from the time of the loss (as other effective programs were already in place in his hospice setting for more recent bereavement grief), and were screened for standard mental health exclusion criteria. Alejandro felt that MBSR was appropriate, but with some key adjustments to better meet this population, such as
	A. Ensuring the class only includes those who were grieving, to create a safe environment where everyone had a shared history
	B. During the teaching on stress physiology, include more on the neurobiology of loss and grief
	C. Adapt some of the poetry to relate more specifically to grief and impermanence
	D. Offer flexibility with the length of meditation practices
	E. Ensure the program is led by a grief counselor within the hospice setting to meet any clinical needs of participants as they arise
	F. Emphasize self-compassion throughout
	• By staying true to MBSR form but making minor shifts to the curriculum, teaching process, and teacher experience, Alejandro felt confident he could draw on MBSR evidence and practice while ensuring it skilfully met this population
2. Does the theoretical premise underpinning mainstream MBPs extend to the population you are considering? If not, what theoretical adaptation is needed?	• MBPs share a theoretical formulation based in ancient wisdom and modern psychology that provides a map of the foundational skills that any MBP curriculum addresses: attention, perspective and self-regulation.^3^ However, sometimes an overlay of a more specific theoretical formulation is required for particular issues or populations. For example, when MBCT for depression was being developed, the challenge was finding cost-effective strategies that enabled people to stay well in the long term. The development process for MBCT sought to use the theoretical formulation of reactivity at times of potential depressive relapse alongside existing CBT strategies (such as behavioral activation and psychoeducation), with intensive training in mindfulness. The rationale was that this would target the mechanisms of depressive relapse more thoroughly than existing approaches had achieved. The evidence has largely borne this out^4^
3. Does the existing MBP curriculum extend to the population and context you are considering? If not, what adaptation (weft) is needed?	• Beyond theory, it is also important to consider both the MBP curriculum and how it is delivered. For example, many settings do not provide two-hour time windows for classes (e.g., schools), so different formats are needed
	• Adaptations may be required to increase MBPs’ reach. For example, when Dr. Brewer, an addictions psychiatrist and mindfulness researcher was leaving work one day, he saw a group of people in the parking lot smoking and looking at their smart phones. Dr. Brewer thought, “If I could bring mindfulness training to them through their smartphones, it could serve so many more people.” He developed an app-delivered MBP for smoking cessation, named Craving to Quit. While much of the learning is asynchronous via a smartphone app, trained MBP teachers are also available to provide live, synchronous mindfulness practices followed by inquiry-based learning for participants to give feedback and guidance on their learning and development. Preliminary and RCT evidence suggests it is acceptable and effective^5^
	• There are many examples where adaptations are necessary to make the curriculum accessible and maximally potent. A UK review of MBPs in healthcare, workplaces, prisons and educational settings provided exemplars, as well as recommendations for research and implementation^6^
4. Do the benefits of an adaptation outweigh the time and costs involved to all in research and implementation? Is the adaptation likely to be sustainable and create long-term value?	• The work on CBT over 50 years is an instructive model. When Beck started this work on CBT for depression there were few evidence-based approaches to depression, let alone other common mental health problems. The case for accessible, evidence-based, scalable psychological treatments was easy to make. Beck and colleagues developed CBT adaptions for anxiety disorder, substance abuse disorders, personality disorders, eating disorders and psychosis.^7^ Each adaptation involved at minimum the publication of a therapist manual, randomized controlled trials demonstrating effectiveness, both against usual care and superiority trials against other treatments, bespoke therapist training and sustained implementation. This extensive and sustained programmatic work means that CBT is now widely available around the world, and in many countries is part of primary care public health service
	• MBPs have gone through their own developmental process. Kabat-Zinn’s original formulation of MBSR was based on a universal model of the mind and body for heterogeneous groups.^35^ Work has focused on its effectiveness and implementation around the world, and there have been numerous adaptations^1,8^
	• But in contrast to CBT, the question of bespoke adaptations came second, with MBCT for recurrent depression perhaps being the most extensively researched^4^ and implemented over the last 20 years. MBCT for recurrent depression is now recommended in clinical guidelines in numerous countries and is becoming increasingly accessible. However, many other MBP adaptations have not been subjected to the same process leaving major research and implementation gaps; arguably this is in part because the time and costs of developing a new adaptation (e.g.,, effort and time to garner research funding, performing research through all stages such as those shown in Table 1, determining if adaption has benefits unique from other existing interventions, dissemination of research findings; scalability of interventions in populations settings, advocacy for policy and health insurance coverage, etc.) were not outweighed by the potential benefits^16^
5. Is there already a good approach for this issue in the population/ context?	• When MBSR was first developed it clearly met a particular need, filling a particular and important niche—helping people in healthcare settings learn to manage and live with long-term health conditions.^11^ Similarly, MBCT for recurrent depression fills a particular need, helping people at risk for depressive relapse learn psychological self-management skills to stay well. There were already a range of evidence-based treatments for current depression. Arguably, some adaptations have not paused to ask this question, “Is there a need or niche for this adaptation?” For example, while MBSR reduces anxiety *symptoms*,^7^ a 2014 systematic review and meta-analyses in participants diagnosed with an anxiety *disorder* showed mixed findings.^58^ Other approaches, most notably CBT, are effective for anxiety disorders, and what is needed now is more sophisticated questions of what works for whom.^59^ It may be that CBT is already a good approach for anxiety disorders, and that mindfulness is not needed. Alternatively, it could be that an adapted MBP might serve this population better by addressing a particular sub-group, offering a more universal preventative approach, or helping patients with particular issues such as working skilfully with strong emotions or improving choices^41^

## How to Adapt an MBP?

**Figure 1.**
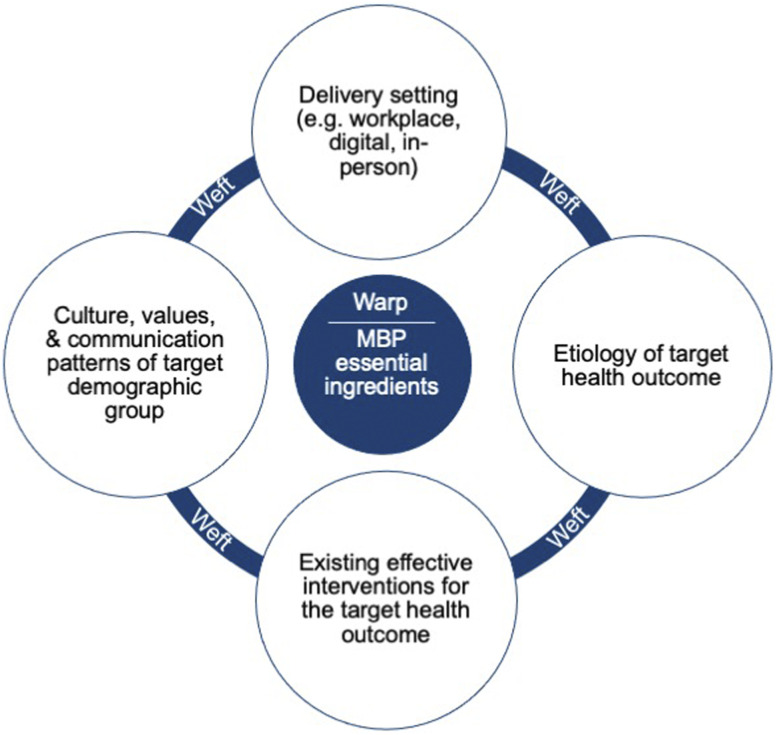
Fundamental knowledge domains required in the developmental team in order to develop an effective MBP adapted to specific populations or contexts.

In order to develop an effective adapted MBP, there are fundamental knowledge domains required in the developmental team (see Figure 1).

### Fundamental Knowledge Domains Needed to Skilfully Adapt an MBP

(1)*Essential ingredients (*i.e., *warp) of MBPs:* It is important to either be, or work with, a highly experienced MBP teacher who has deep knowledge of what MBPs are and aren’t.^37^ MBPs’ warp includes a core theoretical basis that underlines the universal importance of foundational psychological skills such as attention, decentering and self-regulation (see Table 2).^42^ This is an aspect of the essential theoretical ‘DNA’ and does not need adaptation if it is to be considered an MBP. The same applies to some of the vehicles of change within MBPs, namely, experiential learning through core mindfulness practices. These are defining premises of how MBPs are expected to effect change, even if the way they are offered is adapted.
(2)*Etiology of target outcome:* For many conditions and diseases, we know a great deal about what causes, maintains and exacerbates them. For example, blood pressure is sizably affected by obesity, diet, physical activity, alcohol consumption, and antihypertensive medication adherence.^2^ Once we know the modifiable determinants of the health outcome, we can design MBPs to engage participants’ relationships with these, such as through mindful eating or physical activity practices.
In Case Example 2, a theoretical framework was developed using a Theory of Change approach (Table 1) for how mindfulness training could influence blood pressure.^20^ It built upon prior theoretical work by Tang et al.^43^ suggesting that mindfulness impacts 3 domains of self-regulation, specifically attention control, self-awareness, and emotion regulation. The theoretical framework extended to applying these 3 mindfulness skills to people’s relationships with their modifiable determinants of elevated blood pressure, such as diet, physical activity, and antihypertensive medication adherence. Example approaches used in MB-BP were directing participants’ improved *self-awareness* to how they felt before, during and after certain kinds of foods and physical activities, and then being with what insights arose from that awareness. In these scenarios, participants also used their improved *attention control* skills to redirect their attention to healthier choices (e.g., less reactive response to a stressor and healthier food selection).^20,44^ The detailed conceptual framework is described elsewhere.^3,20^
(3)*Existing interventions that work for the health outcome, population, and context:* At times, it may serve the MBP to weave evidence-based elements into extant approaches, in order to maximize efficacy. MBSR itself contains a variety of evidence-based intervention characteristics, such as participatory medicine practices and elements of motivational interviewing, such as during the last class where participants write a letter to themselves setting goals, considering what might get in the way of meeting those goals, and what they will do if that happens.^11^ MBCT integrates MBSR and CBT. A challenge to this approach, particularly from a scientific and mechanistic perspective, is that, as most MBPs are complex behavioral interventions, this affects our ability to understand what the most active ingredients are. Hence, we must balance 2 considerations: Firstly, the encouragement for more dismantling studies using techniques in Table 1 such as the MOST framework to understand the maximally active elements, and cleave out unnecessary elements so that interventions are more cost-effective and efficient. Secondly, as MBP instructors often see during the end of the program when asking participants which practices they will continue with longer term, providing a panel of active elements within the program appears to meet different people in different ways, shown by the diversity of practices that participants plan to practice longer term to support their well-being (e.g., body scan, attentional focus meditation, yoga, and loving-kindness meditation). A sizable range of meditation practices was recently demonstrated in a sample of diverse meditation practitioners,^45^ recognizing different practices support well-being of individuals in unique ways. It is a challenge for mindfulness intervention developers to create interventions that are likely to be accepted by a large segment of society while keeping them efficient and understanding the mechanisms by which they operate.
(4)*Delivery systems and settings:* The variety of settings and methods by which MBPs can be delivered is wide. Delivery can occur in distinct physical settings, such as grade school classrooms, military barracks, corporate workplaces, prisons, or local health centers. MBPs are increasingly being delivered digitally.^26-28^ A frontier of digital mindfulness research is determining the value of these universal elements of MBPs, asking, “What is essential?” For example, Table 2 suggests that 2 essential elements of an MBP are: “Engages the participant in a sustained intensive training in mindfulness meditation practice, in an experiential inquiry-based learning process and in exercises to develop insight and understanding” and the teacher “Is part of a participatory learning process with their students, clients or patients.” Some MBP apps have synchronous (i.e., live) elements with teacher feedback, like Unwinding Anxiety and Eat Right Now.^27,41^ Is the synchronous element needed, and does it maximize positive effects and minimize adverse effects? Alternatively, is purely asynchronous (i.e., static) content, such as that delivered by popular apps like Headspace and Calm, or by books, enough? As technological developments in machine learning, artificial intelligence, social interaction, and mobile sensing continue, there will be increasing opportunities to adapt digitally delivered MBPs in accessible, scalable ways.^46^ Other explorations include stepped care models that would encourage the use of apps, books, self-taught, or lightly supported interventions in mild to moderate conditions—and more intensive teacher/therapist led interventions for people with more challenging issues. There can even be bidirectionality where people may find themselves drawn to different delivery systems at different times in their lives. These are important domains to explore and systematically research as there are sizable accessibility and cost-effectiveness implications.
(5)*Culture, values, and communication patterns of target population:* The importance of culture, race, gender, sexual orientation, and age are increasingly recognized in mindfulness research.^47,48^ In terms of race and culture, a qualitative study by Tenfelde et al, in predominantly low income African American women, found the participants recognized that yoga and mindfulness could be beneficial, and found several recommendations for culturally adapting it to them, including (1) Focusing on stress relief and health benefits; (2) Changing the image of yoga to include the Black community; (3) Peer to peer teaching; and (4) Afro-centric history and connection to yoga.^49^ Bringing forward the culture, values and communication patterns of target groups, whether it is by race, ethnicity, age, gender, or disability status, to name a few, and preferably taught by a member of that group, are important ways to ensure that MBPs are accessible to the broad diversity of people throughout the world, as well as to diverse communities within countries.^47^

For example, in the community-based participatory research being done for the NativeMIND study,^25^ participants are explicitly naming how they are “indigenizing and decolonizing MBSR” as they adapt it to Native American cultures and traditions. This, for example, in one tribal nation, includes using a drum instead of bells to begin and end meditations, and in other tribal nations doing slow ceremonial dances instead of walking meditation. Clear feelings have been expressed in focus groups that NativeMIND is now an authentic expression of their culture and values. When participants feel ‘met’ by the teaching process, the potential for deeper engagement and transformation is significant.^50^ In adapting MBPs, it is fundamental to adapt *with* rather than *for* the communities and contexts.

### Develop and Test Your Adaptation: 8 Steps to Adaptation

Based on experience developing and adapting MBPs, we offer recommendations on steps to adaptation. We recommend researchers follow all steps, while mindfulness teachers may consider following steps 1 through 5. Partnering with researchers is encouraged so that the safety and efficacy of the adaption can be understood, enhancing the chances that the adaptation is evaluated and sustainably implemented. We recommend adhering to best practices for behavioral intervention development, outlined in Table 1. This includes engaging stakeholders in every step of the path (e.g., patients, clinicians, and health insurers) so that an intervention that meets the needs of the population is developed. It is beyond the scope of this paper to outline these steps in detail; a well assembled collaborative team would cover the requisite knowledge and skills to conduct these steps. Refer to the reflective questions in Table 3 while considering these 8 recommended steps for adaption:

(1)*Determine if this work is needed.* Perform a thorough review of the literature, and engage with diverse stakeholders, to establish what is already known about MBPs in the target population and context, and determine if there are already adaptations or alternative non-mindfulness-based approaches being used.
(2)*Articulate the aim and intention of the adaptation, and the theoretical basis of why an MBP would be helpful for this population or context*. Include a theoretical account of the issue of concern in the target population, and how an MBP would address the specific mechanisms (e.g., using the Theory of Change approach in Table 1).
(3)*Generate and develop ideas for the necessary adaptations.* Work creatively with a group of MBP teachers and representative key stakeholders, including those with lived experiences of the issues of concern and the delivery context, and those with expertise in the target population and context.
(4)*Evaluate feasibility:* This can include qualitative interviews with people from the target population/context, single-arm pilot trials of the adapted program monitoring acceptability and feasibility, along with exploratory work about the impact on proposed mechanisms and outcomes.
(5)Steps 3 and 4 continue iteratively until a theoretically plausible, acceptable, feasible, and potentially safe and potent adaptation, is ready to pilot.
(6)*Pilot clinical trial*, with meaningful measures of acceptability, feasibility, harms, adverse events, mechanisms and effectiveness, using a meaningful comparison group.
(7)Perform an adequately powered preregistered randomized controlled trial, adhering to CONSORT guidelines,^51^ to evaluate impacts on the primary outcome of interest, and relevant mechanisms.
(8)If efficacy in step 7 is sizable, carefully proceed with replication, scalability, dissemination, and implementation studies, using stepped implementation science-informed approaches such as those outlined in the NIH Stage Model,^15,16^ Science of Behavior Change,^29^ Obesity-Related Behavioral Intervention Trials (ORBIT) model,^38^ Multiphasic Optimization Strategy (MOST),^52^ and MRC Guidelines on Complex Intervention Development.^30^ as summarized in Table 1.
Our three case examples have progressed through these steps to differing degrees. Case example 1 moved through steps 1–4 to see that a full adaptation of MBSR was not needed, but instead MBSR was used with more minor modifications and screening considerations such as those described in Table 3. To establish if MBCT could be accessible to the general population, the new Finding Peace in a Frantic World (case example 3) passed through steps 1 through 7. It is also emerging in step 8 through book distribution and reader feedback). MB-BP (case example 2) has advanced through steps 1 through 6, with step 7 recently completed and analyses underway.

## Strengths and Limitations of This Framework

Strengths of the MBP adaptation system provided in this paper include grounding it in the established theoretical framework of the essential (warp) and flexible (weft) ingredients of MBPs. The recommendations on how to adapt MBPs are linked to established behavioral intervention development methods such as the NIH Stage Model,^15,16^ Community-Based Participatory Research,^39^ MRC Guidelines on Complex Intervention Development,^30^ and others described in Table 1. Limitations include that, while the warp and weft are informed by theory and practice, the proposed essential and flexible elements have not yet been empirically tested to identify which are more active. These elements were offered by a team of researchers that included some of the first- and second-generation designers of MBSR and MBCT, so reflect their best understanding of active and unique components. Empirically validating the warp and weft elements remains an opportunity for future research.

## Summary and Conclusion

Adapting MBPs is currently happening not only in research, but also by MBP teachers and developers who endeavor to best serve the populations and contexts they work within. This paper provides a set of principles and criteria for when, why and how to adapt MBPs. We suggest ways to ensure adaptations to MBPs are acceptable to populations and contexts, and become potentially scalable, thereby creating efficient and effective programs to maximize public health. Our hope is that this provides a useful framework for ensuring that further developments in the field of MBPs systematically consider safety, acceptability, effectiveness, cost-effectiveness and scalability so their potential to enhance public health and well-being is maximized.

## References

[1] Lee EKP Yeung NCY Xu Z Zhang D Yu CP Wong SYS . Effect and acceptability of mindfulness-based stress reduction program on patients with elevated blood pressure or hypertension: a meta-analysis of randomized controlled trials. Hypertension. 2020;76(6):1992-2001. 33131316 10.1161/HYPERTENSIONAHA.120.16160

[2] Whelton PK Carey RM Aronow WS , et al. 2017 ACC/AHA/AAPA/ABC/ACPM/AGS/APhA/ASH/ASPC/NMA/PCNA guideline for the prevention, detection, evaluation, and management of high blood pressure in adults: executive summary: a report of the American college of cardiology/American heart association task force on clinical practice guidelines. Hypertension. 2017;71(6):1269-1324. 29133354 10.1161/HYP.0000000000000066

[3] Loucks EB Schuman-Olivier Z Britton WB , et al. Mindfulness and cardiovascular disease risk: state of the evidence, plausible mechanisms, and theoretical framework. Curr Cardiol Rep. 2015;17(12):112. 26482755 10.1007/s11886-015-0668-7 PMC4928628

[4] Williams JMG Mindfulness PD . A Practical Guide to Finding Peace in a Frantic World. London: Piatkus; 2011.

[5] Galante J Dufour G Vainre M , et al. A mindfulness-based intervention to increase resilience to stress in university students (the mindful student study): a pragmatic randomised controlled trial. Lancet Public Health. 2018;3(2):e72-e81. 29422189 10.1016/S2468-2667(17)30231-1 PMC5813792

[6] Montero-Marin J Taylor L Crane C , et al. Teachers “finding peace in a frantic world”: an experimental study of self-taught and instructor-led mindfulness program formats on acceptability,effectiveness, and mechanisms. J Educ Psychol. 2021;113(8):1689-1708. DOI: 10.1037/edu0000542. 34912129 PMC8647626

[7] de Vibe M Bjørndal A Fattah S Dyrdal GM Halland E Tanner‐Smith EE . Mindfulness‐based stress reduction (MBSR) for improving health, quality of life and social functioning in adults: a systematic review and meta‐analysis. Campbell Systematic Reviews 2017;13(1):1-264. 10.4073/csr.2017.11.

[8] Kuyken W Warren FC Taylor RS , et al. Efficacy of mindfulness-based cognitive therapy in prevention of depressive relapse: an individual patient data meta-analysis from randomized trials. JAMA Psychiatry. 2016;73(6):565-574. 27119968 10.1001/jamapsychiatry.2016.0076 PMC6640038

[9] Goldberg SB Tucker RP Greene PA , et al. Mindfulness-based interventions for psychiatric disorders: a systematic review and meta-analysis. Clin Psychol Rev. 2018;59:52-60. 29126747 10.1016/j.cpr.2017.10.011 PMC5741505

[10] Khoo EL Small R Cheng W , et al. Comparative evaluation of group-based mindfulness-based stress reduction and cognitive behavioural therapy for the treatment and management of chronic pain: A systematic review and network meta-analysis. Evid Based Ment Health. 2019;22(1):26-35. 30705039 10.1136/ebmental-2018-300062 PMC10270397

[11] Kabat-Zinn J . Full Catastrophe Living: Using the Wisdom of Your Body and Mind to Face Stress, Pain, and Illness. New York, NY: Bantam; 2013.

[12] Herman PM Anderson ML Sherman KJ Balderson BH Turner JA Cherkin DC . Cost-effectiveness of mindfulness-based stress reduction versus cognitive behavioral therapy or usual care among adults with chronic low back pain. Spine. 2017;42(20):1511-1520. 28742756 10.1097/BRS.0000000000002344 PMC5694631

[13] Patten SB Meadows GM . Population-based service planning for implementation of MBCT: linking epidemiologic data to practice. Psychiatr Serv. 2009;60(11):1540-1542. 19880476 10.1176/ps.2009.60.11.1540

[14] Rycroft-Malone J Gradinger F Owen Griffiths H Anderson R Crane RS Gibson A , et al. 'Mind the gaps': the accessibility and implementation of an effective depression relapse prevention programme in UK NHS services: learning from mindfulness-based cognitive therapy through a mixed-methods study. BMJ Open. 2019;9(9):e026244. 10.1136/bmjopen-2018-026244 PMC6738673 31501097

[15] Onken LS Carroll KM Shoham V Cuthbert BN Riddle M . Reenvisioning clinical science: unifying the discipline to improve the public health. Clin Psychol Sci. 2014;2(1):22-34. 25821658 10.1177/2167702613497932 PMC4374633

[16] Dimidjian S Segal ZV . Prospects for a clinical science of mindfulness-based intervention. Am Psychol. 2015;70(7):593-620. 26436311 10.1037/a0039589 PMC5853107

[17] Kristeller JL Wolever RQ . Mindfulness-based eating awareness training for treating binge eating disorder: the conceptual foundation. Eat Disord. 2011;19(1):49-61. 21181579 10.1080/10640266.2011.533605

[18] Bowen S Witkiewitz K Clifasefi SL , et al. Relative efficacy of mindfulness-based relapse prevention, standard relapse prevention, and treatment as usual for substance use disorders: a randomized clinical trial. JAMA Psychiatry. 2014;71(5):547-556. 24647726 10.1001/jamapsychiatry.2013.4546 PMC4489711

[19] Carlson LE Speca M . Mindfulness-Based Cancer Recovery: A Step-by-step MBSR Approach to Help You Cope Wiht Treatment and Reclaim Your Life. Oakland, CA: New Harbinger Publications, Inc.; 2010.

[20] Loucks EB Nardi WR Gutman R , et al. Mindfulness-based blood pressure reduction (MB-BP): stage 1 single-arm clinical trial. PLoS One. 2019;14(11):e0223095. 31774807 10.1371/journal.pone.0223095 PMC6881004

[21] Loucks EB Nardi WR Gutman R , et al. Mindfulness-based college: A stage 1 randomized controlled trial for emerging adult well-being. Psychosom Med. 2021;83(6):602-614. 32947581 10.1097/PSY.0000000000000860 PMC8257475

[22] Jha AP Morrison AB Dainer-Best J Parker S Rostrup N Stanley EA . Minds “at attention”: mindfulness training curbs attentional lapses in military cohorts. PLoS One. 2015;10(2):e0116889. 25671579 10.1371/journal.pone.0116889 PMC4324839

[23] Treleaven DA . Trauma-Sensitive Mindfulness: Practices for Safe and Transformative Healing. New York, NY: W. W. Norton & Company; 2018.

[24] Lönnberg G Jonas W Unternaehrer E Bränström R Nissen E Niemi M . Effects of a mindfulness based childbirth and parenting program on pregnant women's perceived stress and risk of perinatal depression-Results from a randomized controlled trial. J Affect Disord. 2020;262:133-142. 31733457 10.1016/j.jad.2019.10.048

[25] Proulx J. NIH/NCCIH K99/R00 award #5R00AT009570-05. Project Title: Exploring the adaption of mindfulness in native American communities to address diabetes; 2017-2022. https://reporter.nih.gov/search/lOQTtNtk2kGNQnk4DSY5Mw/project-details/10251995

[26] Segal ZV Dimidjian S Beck A , et al. Outcomes of online mindfulness-based cognitive therapy for patients with residual depressive symptoms: a randomized clinical trial. JAMA Psychiatry. 2020;77(6):563-573. 31995132 10.1001/jamapsychiatry.2019.4693 PMC6990961

[27] Mason AE Jhaveri K Cohn M Brewer JA . Testing a mobile mindful eating intervention targeting craving-related eating: feasibility and proof of concept. J Behav Med. 2018;41(2):160-173. 28918456 10.1007/s10865-017-9884-5 PMC5844778

[28] Moulton-Perkins A Moulton D Cavanagh K Jozavi A Strauss C . Systematic review of mindfulness-based cognitive therapy and mindfulness-based stress reduction via group videoconferencing: Feasibility, acceptability, safety, and efficacy. J Psychother Integr. 2020. Advance online publication DOI: 10.1037/int0000216.

[29] Nielsen L Riddle M King JW , et al. The NIH science of behavior change program: transforming the science through a focus on mechanisms of change. Behav Res Ther. 2018;101:3-11. 29110885 10.1016/j.brat.2017.07.002 PMC5756516

[30] Craig P Dieppe P Macintyre S Michie S Nazareth I Petticrew M . Developing and evaluating complex interventions: the new medical research council guidance. BMJ. 2008;337:a1655. 18824488 10.1136/bmj.a1655 PMC2769032

[31] Breuer E Lee L De Silva M Lund C . Using theory of change to design and evaluate public health interventions: a systematic review. Implement Sci. 2016;11:63. 27153985 10.1186/s13012-016-0422-6 PMC4859947

[32] Collins LM . Optimization of Behavioral, Biobehavioral, and Biomedical Interventions: The Multiphase Optimization Strategy (MOST). New York, NY: Springer; 2018.

[33] Sanghvi M Bell R Bristow J Stanway J-P . Fieldbook for Mindfulness Innovators. Sheffield, United Kingdom: The Mindfulness Initiative; 2019. www.themindfulnessinitiative.org.:

[34] McCown D Reibel D Micozzi MS . Resources for Teaching Mindfulness: An International Handbook. Switzerland: Springer International Publishing; 2016.

[35] Didonna F . Clinical Handbook of Mindfulness. New York, NY: Springer Science+Business Media, LLC; 2010.

[36] Dobkin PL Hickman S Monshat K . Holding the heart of mindfulness-based stress reduction: balancing fidelity and imagination when adapting MBSR. Mindfulness. 2014;5:710-718.

[37] Crane RS Brewer J Feldman C , et al. What defines mindfulness-based programs? The warp and the weft. Psychol Med. 2017;47(6):990-999. 28031068 10.1017/S0033291716003317

[38] Czajkowski SM Powell LH Adler N , et al. From ideas to efficacy: the ORBIT model for developing behavioral treatments for chronic diseases. Health Psychol. 2015;34(10):971-982. 25642841 10.1037/hea0000161 PMC4522392

[39] Israel BA Schulz AJ Parker EA Becker AB . Review of community-based research: assessing partnership approaches to improve public health. Annu Rev Public Health. 1998;19:173-202. 9611617 10.1146/annurev.publhealth.19.1.173

[40] Griffith GM Bartley T Crane RS . The inside out group model: teaching groups in mindfulness-based programs. Mindfulness. 2019;10:1315-1327.

[41] Roy A Druker S Hoge EA Brewer JA . Physician anxiety and burnout: symptom correlates and a prospective pilot study of app-delivered mindfulness training. JMIR Mhealth Uhealth. 2020;8(4):e15608. 32234708 10.2196/15608 PMC7160707

[42] Feldman C Kuyken W . Mindfulness: Ancient Wisdom Meets Modern Psychology. New York: Guilford; 2019.

[43] Tang YY Hölzel BK Posner MI . The neuroscience of mindfulness meditation. Nat Rev Neurosci. 2015;16(4):213-225. 25783612 10.1038/nrn3916

[44] Nardi WR Harrison A Saadeh FB Webb J Wentz AE Loucks EB . Mindfulness and cardiovascular health: qualitative findings on mechanisms from the mindfulness-based blood pressure reduction (MB-BP) study. PLoS One. 2020;15(9):e0239533. 32966308 10.1371/journal.pone.0239533 PMC7510988

[45] Matko K Ott U Sedlmeier P . What do meditators do when they meditate? Mindfulness. 2021;12:1791-1811.

[46] Gál É atefan S Cristea IA . The efficacy of mindfulness meditation apps in enhancing users’ well-being and mental health related outcomes: a meta-analysis of randomized controlled trials. J Affect Disord. 2021;279:131-142. 33049431 10.1016/j.jad.2020.09.134

[47] Proulx J Croff R Oken B , et al. Considerations for research and development of culturally relevant mindfulness interventions in American minority communities. Mindfulness (N Y). 2018;9(2):361-370. 29892321 10.1007/s12671-017-0785-z PMC5992912

[48] Carlson LE . Uptake of mindfulness-based interventions: a phenomenon of wealthy white western women? Clin Psychol. 2018;25(3):e12258.

[49] Tenfelde SM Hatchett L Saban KL . “Maybe black girls do yoga”: a focus group study with predominantly low-income African-American women. Complement Ther Med. 2018;40:230-235. 30219456 10.1016/j.ctim.2017.11.017

[50] Lakey G . Facilitating Group Learning: Strategies for Success with Diverse Adult Learners. San Francisco, CA: Jossey-Bass; 2010.

[51] Boutron I Moher D Altman DG Schulz KF Ravaud P . Extending the CONSORT statement to randomized trials of nonpharmacologic treatment: explanation and elaboration. Ann Intern Med. 2008;148(4):295-309. 18283207 10.7326/0003-4819-148-4-200802190-00008

[52] Collins LM Murphy SA Nair VN Strecher VJ . A strategy for optimizing and evaluating behavioral interventions. Ann Behav Med. 2005;30(1):65-73. 16097907 10.1207/s15324796abm3001_8

